# Cross-PLC: An I3oT Cross Platform to Manage Communications for Applications in Real Factories

**DOI:** 10.3390/s25102973

**Published:** 2025-05-08

**Authors:** Antonio Lacasa, Javier Llopis, Nicolás Montés, Ivan Peinado-Asensi, Eduardo Garcia

**Affiliations:** 1 Ford Spain, Poligono Industrial Ford S/N, 46440 Almussafes, Spain; alacasa1@ford.com (A.L.); jllopis4@ford.com (J.L.); egarci75@ford.com (E.G.); 2 Department of Mathematics, Physics and Technological Sciences, University CEU Cardenal Herrera, C/San Bartolome 55, 46115 Alfara del Patriarca, Spain; ivan.peinadoasensi@uchceu.es

**Keywords:** IIoT, I3oT, cross platforms, Cross-PLC, Industry 4.0

## Abstract

Recently, a new concept has emerged for the development of Industrial Internet of Things (IIoT) applications, the Industrializable Industrial Internet of Things (I3oT). As a criterion for the design of industrial applications, the I3oT imposes the exclusive use of pre-installed elements in the company such as PLCs, sensors, IT/OT networks, etc., trying to minimize the impact on the factories and guaranteeing a cheap and assumable scalability for companies, something that cannot be implemented with the vast majority of IIoT applications available in the market. In our previous work, we have used I3oT applications for predictive maintenance on different components: cylinders, presses, welding clamps and also energy-saving tools, detection of bottlenecks and sub-bottlenecks, etc., all of them generalized for the entire factory. However, the main drawback comes from the flow of data through the IT/OT network. This article presents the Cross-PLC, a tool to allow massive data extraction using the company’s IT/OT network by communicating with any type of PLC or brand existing in the market. The Cross-PLC performs passive listening, and through different communication criteria, the Cross-PLC becomes a virtual PLC containing all the parameters necessary for the I3oT applications developed. This article presents the design of this tool, its implementation and use at Ford Factory in Almussafes (Valencia).

## 1. Factory Conditions in the Fourth Industrial Revolution: Welcome to “The Jungle”

The governance of factories in the automotive sector is extremely complex. These factories use thousands of robots, clamps, cylinders, conveyor belts, etc., equipment with its components, electric motors, gears, chains, with each of the elements applied to different processes, such as welding, stamping, painting, etc. In addition, all this machinery interacts with the operators involved in different phases of the process, who work assembling the components, verifying the quality of the parts, and in some cases, they must modify machine parameters to guarantee the productivity and quality of the parts. The goal of automation is none other than to try to eliminate dependence on that human factor. However, some machines are not able to adapt to all plant situations, which calls into question whether a complete automation of a factory without human presence would be the most efficient approach, see, for example, [1,2]. With the emergence of Industry 4.0 and its technologies: Industrial Internet of Things (IIoT), Big Data, Digital and Hybrid Twins, etc., a significant improvement in factory governance is expected in which data will become a lens through which Industry 4.0 can be constructed [3]. The Industrial Internet of Things (IIoT) is one of the most relevant enabling technologies of the Industry 4.0 (I4.0), [4,5] and consists of the application of the IoT in the industry. Namely, the IIoT is applied to connect machines and devices in industrial environments, focusing on machine-to-machine communication; any failure can lead to high-risk losses and the amount of data collected in IIoT is much larger than in IoT [6]. Furthermore, IIoT is also considered an I4.0 equivalent concept in some of the literature [7].

When the industry or the responsible managers have to decide whether to install IIoT applications in their company on a massive level, they are often discouraged by several factors and these are as follows:

- Energy efficiency: since most IIoT devices are powered by batteries [8,9,10].
- *Interoperability*: since connecting so many devices is usually a serious challenge for the IIoT [9].
- *Safety*: since information and privacy are vital at the corporate level [6,8,9,10].
- *Scalability*: since the massification of IIoT solutions implies the use of a huge number of devices connected to each other in hierarchical subdomains [9].
- *Maintenance and updates*: since system operators will not only have to manage the original system but also all the new ones, and therefore, many engineers will have to be trained for this [6,9].
- *IT/OT integration*: IIoT systems require the convergence of OT and IT for the integration of data from both parties [6].
- *Cultural change*: Most of the industries resist change because they are afraid and do not understand the technology associated with IIoT [6].

There is an important effort in the literature to overcome the previous issues. The research on functional architectures is a trend in automation and supervision applied to I4.0- and IIoT-enabled factories [11,12,13,14,15,16,17,18]. An interesting conclusion was obtained in [11] because experiment-based architectures are increasing over time to overcome the actual problems. In ref. [18], states that implemented IIoT applications are scarce, and most of the architectures are hypothetical, studied using software simulations, virtual environments or use cases providing scarce implementation information, which are scenarios that are far from real applications for industrial processes.

The transition from the current installation configuration of industrial production governed by traditional industrial control systems (ICS) to a configuration of Industry 4.0 requires a progressive transformation. It involves an adaptation of the devices and architectures used in ICS to adapt to the new paradigm in order to achieve both vertical and horizontal integration of devices at all levels [16]. One of the industrial elements that has been key throughout the automation revolution is the programmable logic controller. Its role has been mainly to command low-level configurations providing a first layer of control for the processes and being the closest interlocutor to both the sensors and the actuators. Recently, proposals have been made for the development of new PLCs for Industry 4.0. such as IoT-PLC, see [19], or PLC 4.0, see [20]. The first PLC for IIoT was developed by the company Phoenix Contact, Telford, Shropshire.

However, the updating of the existing elements in the factories is usually slow. In automotive factories, we can find thousands of robots, grippers, welding clamps, conveyors, elevators, etc., that are part of the manufacturing lines, each one of them sensorized and governed mostly by PLCs. Companies have, as one of their objectives, to make their assets profitable, and for this reason, old and new technologies of the last 20/30 years had to coexist within large factories, and therefore, the update is only carried out when obsolete elements stop working or there is an urgent need to update them. This mixture of technologies and brands, together with the unpredictability of breakdowns, the consequent line stops, loss of production and the need to guarantee the daily production ratio, is what makes today’s factories known as “the jungle”.

### 1.1. Previous Works: I3oT (Industrializable Industrial Internet of Things)

In order to generate IIoT solutions that are able to adapt to the reality of the factories and the needs of the IIoT tools described, we have proposed in our previous works, see [21], a new concept: the Industrializable Industrial Internet of Things (I3oT). The approach of this new concept is the use of the installations available in the factories to develop IIoT applications from them. The machines installed in the industry operate automatically and have sensors that provide the information received by the PLC to control the lines. The factories have an IT/OT network through which the machines communicate and the factory is managed. Under this paradigm the *I3oT* applications could be easily extrapolated and scalable to the rest of the systems at a very low cost, allowing the definitive establishment of Industry 4.0 and its technologies. The following sub-sections show the different tools developed so far.

#### 1.1.1. Miniterms

One of the first I3oT applications developed were the so-called *Mini-terms,* and these were described in ref. [22]. The mini-terms are based on programming a timer in the PLC or PC-Line so that through the sensors installed for the normal operation of the line, we can measure the time it takes for the line elements to perform their task. These data are sent through the OT and IT networks for analysis and processing. Deterioration over time is an indicator that the component is near the end of its useful life and it may produce a line stoppage. The great advantage of using the *mini-terms* is that there is no need to install new sensors, and their industrialization is immediate. Currently, at Ford factory in Almussafes (Valencia), there are more than 46,000 elements or components under surveillance with this technology that detect anomalies in pneumatic and electrical welding clamps, screwdrivers, clamps, elevators, etc. The use of mini-terms to anticipate machine failure is generating important benefits at an industrial level, such as the increase in technical availability (TAV), >6%, see [22].

#### 1.1.2. Criterion C-360

In ref. [21], it is proposed to use the *I3oT* concept in the stamping process through the so-called *Criterion C-360*. With this criterion, 360 values per cycle are generated from the variables or sensors available in the stamping process. Unlike the use of *mini-terms*, there are 360 values per cycle, while in the *mini-term*, there was one value used per cycle, which allows us to evaluate the possibilities of the I3oT philosophy when applied to other industrial processes. Many applications that the (I3oT) system may have in the stamping process, and in particular, of *Criterion C-360*, are yet to be discovered. Based on this criterion, many applications have been developed for predictive maintenance based on tonnage sensors [23], another tool used for energy saving [24] and finally, the feasibility of developing a digital twin for the stamping process is explored through the *I3oT* philosophy of the stamping process [25]. The following sub-sections show more information about these applications.

#### 1.1.3. Predictive Maintenance on Presses: Gravity Center (GC)

One of the ways to monitor the press health is checking that the effort made by the press is carried out in the most balanced way. In the tool developed in ref. [23], the tonnage variables are used to calculate the gravity center of the moving part of the press; the slide and the die that move through the guides located in the four columns of the press. The system calculates the 360 values of the gravity center for each stroke, and when the center goes out of the set limits, an alarm is generated to alert the maintenance operators that something anomalous is happening, see [23].

#### 1.1.4. Manufacturing Maps

In ref. [26], the use of the Petri Nets together with *mini-terms* is proposed to model the factory by layers in order to build a factory model through the *manufacturing maps*. These maps are considered the “Google Maps” of a factory. On the top level we have the *commodity view*, a complete view of the factory whose level would be more focused on senior managers and then, we go down layer by layer, passing through the *line view* and *station view* until reaching the machine level, where we will directly have the *mini-terms*. The *manufacturing maps* have two fundamental components, Big Data based on *mini-terms* and Petri nets. Big Data provide the sub-cycle time data of the components and Petri nets provide the complete modeling of the factory that allows us to reconstruct the information based on *mini-terms* at any level, see [26]. In ref. [27], the author uses *manufacturing maps* to detect bottlenecks and sub-bottlenecks and propose improvements to the production line in order to reduce energy consumption and/or increase jobs per hour (JPH).

#### 1.1.5. Secondary Wear Detection on Welding Clamps by Virtual Sensors

Another I3oT application developed in our previous work was the detection of secondary wear in welding clamps. This is critical for maintaining the efficiency and quality of welding operations. The secondary part, which is responsible for transferring the electrical energy from the transformer to the electrode, is subject to constant wear due to the heat and pressure during the welding process. The detection of secondary component wear is therefore essential to avoid potential issues such as increased energy consumption, decreased quality and frequent downtime. In ref. [28], the author discusses the importance of monitoring secondary wear, highlighting the importance of keeping accurate records of secondary wear, and replacing parts when necessary to avoid unexpected downtime.

#### 1.1.6. Energy Savings on Production Lines

As mentioned in the introduction, the pressure to which the operators are subjected limits them from optimizing certain parameters of the lines because they prefer not to take a risk in order to avoid consequences. We can find a clear example of this problem when choosing the speed of industrial robots. In large factories such as Ford Valencia, there are thousands of robot arms that are usually placed by default at 100% of their maximum speed, which generates unnecessary energy consumption. In ref. [29], the author seeks to develop an algorithm to reduce the speed of the robot arms automatically so that energy saving will be generated. The level of energy savings depends on several factors, but the most influential one is line imbalance, something that is usually more common in the lines that produce different car models and variants. In ref. [29], the simulation of the manufacturing lines was used to estimate energy savings managing to reduce consumption by 57%. Moreover, in ref. [29], this simulation was used with a real mono-model line achieving a saving of 11%.

### 1.2. Cross Platforms

The cross-platform software (also called multi-platform or platform-independent software) is a computer software designed to work across multiple computing platforms. Some cross-platform programs require a separate compilation for each platform, but others can run directly on any platform without any special preparation. It is mainly used for the development of applications for mobile phones, and it is important that a particular app can be used for any platform. In ref. [30], a study was carried out on the apps available in the Google Play Store in order to determine how many of them had been developed with cross-platform software. The study indicates that 15% of the total has been developed using this technology. The advantages of working with this software is the use of uniform designs, the reduction in time and cost in production and the implementation of faster and more flexible updates. Conversely, we find performance limitations as we do not have access to native features and we always have platform dependency [31].

The development of cross-platform applications is also extended to other areas of engineering. For example, we can find cross-platform applications for programming robots [32,33,34] or also for programming computer numerical control machines (CNC) [33] and even developing a cross-platform application to manage communications at the industrial level [35] or programing PLCs with a cross-platform application such as PLC-PROG [36].

### 1.3. Purpose of This Article

The objective of this article is to develop a cross-platform *I3oT* tool to extract information from various PLCs and their variants and generations co-existing in a factory in the least intrusive way possible. This tool has been called *Cross-PLC* and will be the tool to which all *I3oT* applications will be connected to manage their communication with the factory PLCs.

The main handicap for the *I3oT* philosophy to grow in more monitored elements and new developments lies in the IT/OT networks available in the companies and in the diversity of programmable elements (PLCs) that we can find in factories of the automotive sector. In order to be less intrusive but more efficient and scalable, the passive method and the actuator model will be used to communicate with the PLCs.

As seen in our previous works, the *I3oT* concept and then *Cross-PLC* allows us to develop IIoT applications with low cost and industrial impact, eliminating or minimizing all the problems reported in the literature about the IIoT applications regarding energy efficiency, security, scalability, maintenance and updates, IT/OT integration and cultural change [6,8,9,10].

The organization of the remainder of the paper is as follows. The *Cross-PLC* platform design is defined in Section 2. Section 3 defines the implementation of the *Cross-PLC* platform, and Section 4 shows some experimental results in the Ford Factory in Valencia with PLC’s in Siemens and Rockwell. Section 5 and Section 6 show the discussion and conclusion, respectively.

## 2. Design of the Cross-Platform *I3oT* Tool, *Cross-PLC*

**Figure 1:**
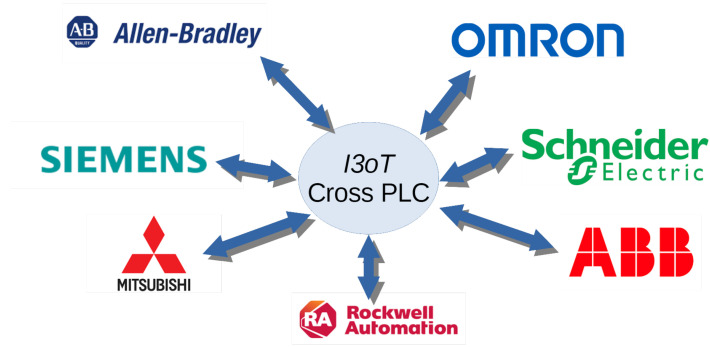
*I3oT* cross platform, *Cross-PLC*.

The objective of the *I3oT* cross-platform design, *Cross-PLC*, is the development of an application that facilitates communication with the programmable logic controllers (PLCs) that allows information to be extracted from them with the least possible impact on the pre-installed systems in the factory. Figure 1 shows the concept of *Cross-PLC*.

### 2.1. Choosing the Communication Method

Communication in computer systems can be classified into two main types; active and passive. In active communication, the sender initiates and controls the communication, sending data to a receiver on a regular basis or when necessary according to a predefined schedule. For example, in a temperature monitoring system in a warehouse, the temperature sensor is checked every minute by a monitoring application. The advantages of active communication are as follows:

1. *Precise control*: the sender has complete control over when and what data are sent.
2. *Regular updates*: the sender provides regular updates, which can be useful for applications that require frequently updated data.

Disadvantages of active communication:

1. *Network saturation*: when there are too many devices sending data frequently, it can cause network congestion.
2. *Power consumption*: on mobile devices or embedded systems, constant active communication can consume more battery power.
3. *Potential latency*: data may not be immediately available when needed as they depend on the predefined time interval for querying the data.

In contrast, in passive communication, the receiver initiates communication when it is ready to send data and the sender simply detects and responds to requests. For example, in a sensor system in a production plant the sensors will only send data when they detect a significant change in plant conditions. The advantages of passive communication are as follows:

1. *Network efficiency*: avoids network saturation by sending data only when needed.
2. *Energy saving*: reduces energy consumption by avoiding constant communication.
3. *Quick response*: provides a quick response to requests as data are sent immediately when available.

For the communication development of the *I3oT Cross-PLC* tool, passive communication is better than active communication in several crucial aspects. First, its efficiency in managing network resources is remarkable. By sending information only in response to specific events, passive communication minimizes data traffic, thus avoiding network congestion and ensuring optimal use of available resources. On the other hand, active communication has to make continuous requests at regular time intervals until it can obtain the expected value, which can generate a greater volume of traffic, which, in turn, could potentially saturate the network and slow down the performance of the system. Another point to note is that passive communication demonstrates superior responsiveness to specific events and large frames. By allowing the devices to respond only to relevant events, the waiting time is significantly reduced and the responsiveness of the system is improved; if, for example, 5000 data per frame are to be sent, it is more effective if the data source sends them when it knows that its data have changed, and therefore, it does not need to be requesting every x time interval if any of those 5000 data have changed. This is in contrast to active communication that has a predefined response time and is less flexible.

### 2.2. Cross-PLC Actuator Model

The actuator model was proposed by Carl Hewitt in 1973 as a way to address concurrency in distributed applications. This paper proposes an approach to modeling systems as a collection of interacting actuators.

In the actuator model, the actuators are independent computational entities that can run concurrently and asynchronously. Each actuator has its own internal state and can communicate with other actuators by sending them messages. This ability to communicate between actuators is essential to build distributed systems in which multiple components must interact with each other efficiently and scalably. In distributed applications with a high demand for connections, the actuator model offers a flexible and modular architecture. Actuators can process messages independently, allowing for non-blocking concurrency. This means that an actuator does not have to wait for another actuator to finish its task before continuing with its own, which avoids blockages and bottlenecks in the execution of the program. In addition, actuators can be easily scaled by adding more instances of actuators to distribute the workload, ensuring high scalability of the system.

The actuator model offers several key advantages for distributed applications with a high demand for connections. First, it provides effective isolation and modularity as each actuator is an independent entity with its own internal state and behavior. This facilitates the construction of modular systems and the management of the status in a clear and controlled manner. Second, non-blocking concurrency enables efficient message processing, ensuring optimal performance even under heavy workloads. Third, the inherent scalability of the actuator model allows the system to easily grow and handle a higher volume of connections without sacrificing performance. In addition, fault tolerance is improved due to the ability to monitor and recover between actuators, which guarantees the robustness of the system. Finally, the flexibility of the actuator model allows for easy adaptation and evolution of the system as user requirements and demands change.

### 2.3. Network Architecture in Cross-PLCs

Cross-PLCs focus on efficient and versatile communication with PLCs, taking advantage of the native communications of the different PLCs available on the market: Rockwell, Siemens, OMRON, ABB, Schneider, Allan Bradley, etc., using specific protocols of each of them to maximize compatibility and efficiency in communication. The actuator model allows the Cross-PLC application to develop according to the communication needs and the different brands existing in the factory. By way of example, the following subsections show how communication has been carried out for the two most used PLCs, Rockwell and Siemens, at the Ford factory in Almussafes, (Valencia, Spain).

#### 2.3.1. Communication with Rockwell PLCs in Cross-PLCs

Communication with Rockwell PLCs is performed using the common industrial protocol (CIP), an industry communication standard widely used in Rockwell devices. CIP is an object-oriented, ethernet-based protocol that provides a common platform for data communication, control and configuration in industrial environments. This protocol offers a wide range of services including process data exchange, remote programming and device status monitoring. CIP is designed to be scalable and flexible, allowing the integration of devices from different manufacturers within the same system and facilitating interoperability among industrial automation equipment.

#### 2.3.2. Communication with Siemens PLCs in Cross-PLCs

Communication with Siemens PLCs is carried out through the ISO protocol (https://www.rfc-editor.org/rfc/inline-errata/rfc1006.html, accesed on 28 April 2025) over transmission control protocol (TCP), a choice that guarantees compatibility with the devices of this manufacturer. The ISO protocol over TCP is an implementation of the ISO 8073 standard, which defines a set of rules for communication between distributed systems over TCP/IP networks. This protocol provides a mechanism for the reliable and efficient exchange of data using TCP connections to ensure the orderly and error-free delivery of messages. ISO over TCP offers features such as message segmentation and reassembly, flow control and error detection and recovery, making it suitable for industrial applications where reliability and data integrity are critical.

#### 2.3.3. Bases and Fundamentals of the Implemented Network Architecture

The architecture of this application is based on fundamental principles of industrial communication and modular and scalable design approaches that make the application simulate a virtual PLC. This is achieved through an object-oriented design that encapsulates the logic of communication and control in reusable and easily configurable components. This allows seamless integration with different devices and systems regardless of their manufacturer or communication protocol. The different components of the application such as communication modules, data management services and user interfaces are implemented as independent actuators that can be scaled and adapted according to the needs of the system.

#### 2.3.4. Sending Data to MQTT Architecture

Message queuing telemetry transport (MQTT) is a lightweight, open-source messaging protocol designed for communication between devices on networks with limited bandwidth or unstable connections. The MQTT version used in the present paper is V 3.1.1.1. This protocol is based on the publish/subscribe model, where devices can post messages on a “topic” and subscribe to topics to receive relevant messages. RFC9431 https://www.rfc-editor.org/rfc/rfc9431.pdf, (accessed on 28 April 2025), RFC 9431 https://www.rfc-editor.org/rfc/rfc9431.pdf, (accessed on 28 April 2025) RFC 7252, https://www.rfc-editor.org/rfc/pdfrfc/rfc7252.txt.pdf, (accessed on 28 April 2025). This model is chosen as the final part of the Cross-PLC architecture. Once the data are obtained from the PLCs, they are sent through this protocol, which has three main components: the client, the broker and the publisher/subscriber, with Cross-PLC being the publisher. Some of the main advantages of using MQTT are as follows:

1. *Bandwidth usage efficiency:* MQTT uses a compact message format, minimizing bandwidth consumption and enabling efficient communication even on networks with bandwidth limitations.
2. *Scalability:* MQTT’s publish/subscribe model allows for flexible scalability, as clients can subscribe to multiple topics, and brokers can handle a large number of clients and messages simultaneously.
3. *Reliability:* MQTT offers integrated delivery confirmation and quality of service (QoS) mechanisms to ensure the reliability of message delivery, making it suitable for applications in which data integrity is critical.
4. *Flexibility:* MQTT is highly adaptable and can be deployed on a wide variety of devices and platforms from resource-constrained embedded devices to high-capacity servers, making it suitable for a wide range of Internet of Things (IoT) applications, telemetry systems, instant messaging and more.
5. *Compatibility:* MQTT is a standard and open source protocol with implementations available in a variety of programming languages and platforms, ensuring interoperability and ease of integration with existing systems.

### 2.4. Cross-PLC Concept with PLC ControlLogix Communications

The developed Cross-PLC system accepts PLC communications and transforms them to a JSON structure to store and analyze data obtained from the factory. In the case of Rockwell, it exchanges PLC data via unconnected CIP (https://datatracker.ietf.org/doc/html/rfc2653, accessed on 28 April 2025) Data Write Tables Messages with MQTT Servers. empowering PLC as an event-driven model and reducing network bandwidth usage. The currently supported features are as follows:

- CIP Write Data Table. Only basic types SINT, INT, DINT, REAL.
- Accept any remote element. *Can be overwritten by any PLC.*
- Fragment CIP Write. More than 104 DINT elements.
- Simple CIP Read Data Table Messages. Only basic types SINT, INT, DINT, REAL.
- Simple CIP Write Data Table Messages for Structures parsed as SINT.
- Simple CIP Read Data Table Messages for Structures.
- API swagger-ui at api-docs.
- Web page.
- Metrics.

For this, a structure has been created in which we receive data from the connections of the PLCs to the server. An active connection from a PLC to the Cross-PLC is a network card that has sent data in the last 30 s. A card in Rockwell can be used to send data from multiple PLCs, and each connection has a TAG associated with it. A TAG refers to the “Destination Element” of the MSG instruction of the Rockwell PLCs. It is a variable of the virtual PLC so it cannot start with a number; it must start with letters or low dash.

**Figure 2:**
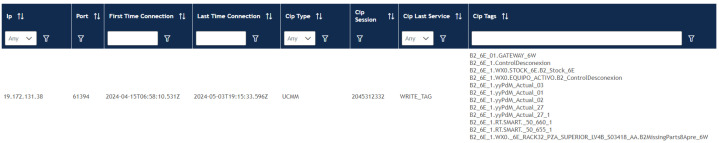
Example of an active connection with its TAGS.

In Figure 2, we can see a connection from a card with its IP address that has several TAGS associated with it that correspond to a PLC data sending block. TAGS are classified into two types, stored TAGs and active TAGs. Active tags are those that have received data within 30 s, while stored tags are those that have been written (and read) and may not be counted in active tags because they have not been sent again after 30 s.

**Figure 3:**
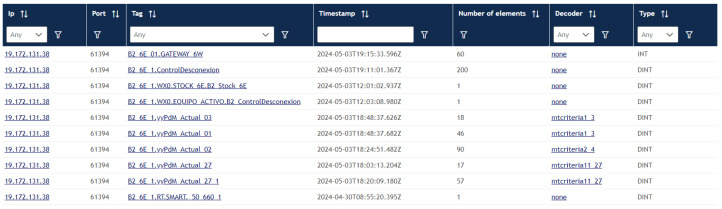
List of active tags.

**Figure 4:**
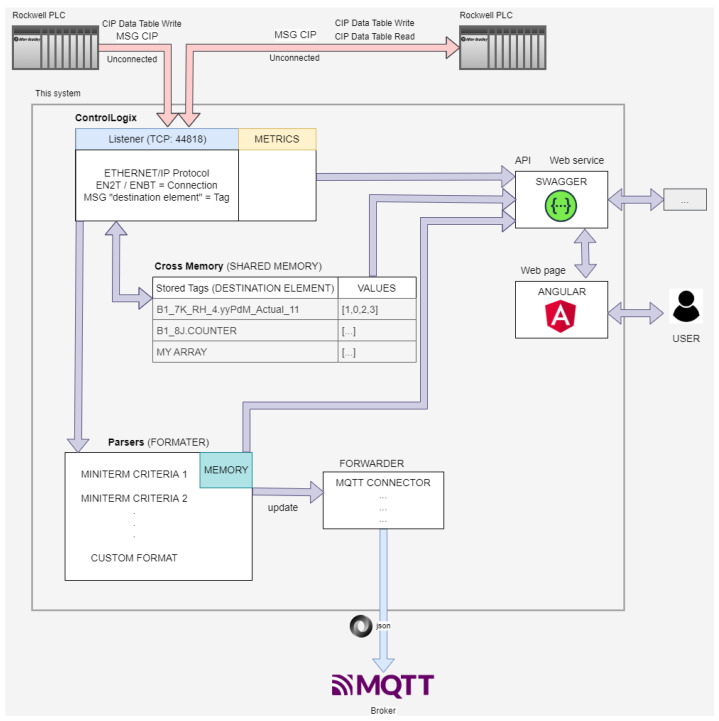
Cross PLC Architecture for ControlLogix.

Figure 3 shows the active tags that have sent information to the server within the last 30 s, being able to know which links and connections are working. It also shows the number of elements associated with each tag and the type of data being received. For each tag, a parser or formatter is associated that indicates the method in which the structure blocks are decoded before having support and being sent to the MQTT user, see Figure 4.

#### 2.4.1. Criteria Designed for Communication with Various Line Components

In order to adjust the data or requirements of the applications to the transfer of information, the Cross-PLC allows the creation of criteria that make up data packets. These criteria are programmed into the PLC that packages the information and transmits it with this specific criteria. There are currently six types of criteria defined to identify the behavior of mechanical elements in terms of movement time and the physical parameters measured on the machines. As the I3oT concept develops in more specific applications, it is expected that the number of criteria will increase. The criteria currently defined with the applications developed are as follows:

- **Criterion 01:** a device that requires a single measurement of the mini-term to capture the entire operation. For example, a robotic water gun, lifting device or safety window.
- **Criterion 02:** a device that requires multiple mini-term measurements to capture the entire operation. For example, a pneumatic valve that actuates multiple clamps.
- **Criterion 03:** a device that requires a single mini-term measurement and travels in only one direction within a given station. For example, a clamp on a pallet with a “docking” station so that it is closed in the first station but open in the last one.
- **Criterion 04:** a device that requires multiple mini-term measurements and travels in a single direction within a given station. For example, a group of grippers on a pallet with a “docking” station that is closed in the first station but open in the last one.
- **Criterion 11:** routine to collect a single measurement of the mini-term and the physical parameter. For example, a servo-actuated robotic welding gun. The robot measures time and current and then sends the results to the PLC (more accurate method).
- **Criterion 360:** specific to presses, requiring 360 measurements of a physical parameter of the press that are traveling in only one direction, for example, any physical parameter obtained from presses such as: tonnage, speed, pressures, temperatures, consumptions, strength, etc.

#### 2.4.2. Memory Usage

All the numbers below are from a 1756-L73S processor running V20.18 firmware.

Loading all required routines, Tags, UDTs and with one (1) Criterion 01 and one (1) Criterion 02 module, then the memory usage is 27072 Bytes.

After the initial memory usage above, then each call/request for a Criteria 01 routine requires 272 Bytes.

After the initial memory usage above, then each call/request for a Criteria 02 routine requires 632 Bytes.

Scan Time All the numbers below are from a 1756-L73S processor running V20.18 firmware. Based on adding three (3) Criterion 01 devices, the scan time is increased by 3.174 milliseconds.

Then, adding one routine, it increases by 0.001 milliseconds.

## 3. Implementation

### 3.1. ControlLogix (Listener Driver)

The Cross-PLC accepts any ControlLogix CIP MSG Data Table Write Unconnected (fragmented or not) and parses the Tags or the destination element to a json. The system can respond to a limited CIP Data Table Read Unconnected Messages if the element to read exists in the shared memory. If the tag is not present in shared memory, an *“ERROR: EPATH Invalid Parameter (0x20) + destination not assigned (0x01)”* message is sent to the requester PLC.

The data types accepted are arrays (any size) of the following:

- SINT
- INT
- DINT
- REAL

If any other type or structure is sent, then an *“ERROR: EPATH Invalid Parameter (0x20) + destination unknown (0x00)”* message will be sent to the requester PLC. Connections will be dropped after the OS timeout TCP connection time is achieved (normally 30 s).

### 3.2. CrossMemory (Shared Memory)

In this part, all received tags (destination element) are stored on RAM memory. It is used to reply to the Read Data Table request and API services. This memory does not take care about who write the tags. It means that any write from any PLC to the same tag name will be overwritten and stored in the same place (“whiteboard”).

### 3.3. Parsers (Transformation)

This part takes care to transform the array of data to a json structure. It receives any update of CrossMemory tags if then they match with the pattern of the tagname (“Destination element”) of the parser. We also have to take care about duplicates and store our own dictionary of sent messages. The output of the parser is sent to the forwarder to be processed.

### 3.4. Sending (Forwarding)

This component sends the parser output ot the MQTT server/broker as a json message. An example for the mini-terms parser is as follows:

Routing key:

- mt.factory02.area01.(_a1_l8_1)_actual_02.114.1

{

‘‘device_criteria’’: 2,

‘‘device_type’’: 5,

‘‘trigger_value’’: 145279,

‘‘value’’: 867,

‘‘job_id’’: 1,

‘‘tag_position’’: 114,

‘‘host’’: ‘‘host01’’,

‘‘plant’’: ‘‘area01’’,

‘‘date’’: ‘‘2024-09-03T07:12:05.009Z’’,

‘‘topic_id’’: ‘‘factory02.area01.(\_a1\_l8\_1)\_actual\_02.114.1’’,

‘‘mqtt_routing’’: ‘‘mt.factory02.area01.(\_a1\_l8\_1)\_actual\_02.114.1’’,

‘‘factory’’: ‘‘factory02’’

}

An example for the generic parser is as follows:

Routing key:

- factory01.area15.manuf_line01.SCA_WARNING

{

‘‘tagname’’: ‘‘manuf\_line01.SCA\_WARNING’’,

‘‘pos_dest’’: 0,

‘‘type’’: 196,

‘‘type_txt’’: ‘‘DINT’’,

‘‘type_size’’: 4,

‘‘number_of_elements’’: 1,

‘‘timestamp’’: ‘‘2024-10-26T14:19:14.983Z’’,

‘‘timestamp_end’’: ‘‘2024-10-26T14:19:14.983Z’’,

‘‘values’’: [1],

‘‘ip_form’’: ‘‘19.100.100.10’’,

‘‘port_form’’: 1234,

‘‘CIP_session’’: 4023284046,

‘‘CIP_context’’: ‘‘13aca2430000e414’’,

‘‘decoderMsgsEmitted’’: 23,

‘‘host’’: ‘‘host01’’,

‘‘factory’’: ‘‘factory01’’,

‘‘plant’’: ‘‘area15’’

}

The “routing key” to the MQTT server is the combination of the information regarding the “factory”, “plant” and “tagname”.

- factory: is configured at config file.
- plant: is configured at config file.
- tagname: is the “Destination element”.

- Example: 0145a.body1.B1_7X_LH_7.RT._010R2SCA_BARREL_WARNING

Also add “mt” as the first key if it is mini-terms parser activated and contains the special structure of *tagname.position.jobid* with adding brackets at the first part of tagname, taking out the dot.

- Example: mt.0097a.body1.(_b1_8c_c519_1)yypdm_actual_02.115.1

### 3.5. Config File

On the deploy process, a config file is placed at the same level of the binary file. It is a JS file with the next configurable parameters:

- EIP_PORT: TCP port for listening CIP commands (default 44818).
- HOST_EIP_BIND: IP of host network card to limit listening of CIP messages (0.0.0.0 to read on all network cards).
- PLANT: Prefix for MQTT topic routing.
- FACTORY: Prefix for MQTT topic routing.
- API_WEB_PORT: Port for listening to API web services and web page.
- HOST_API_WEB_BIND: IP of host network card to limit listening to API web services and web page (0.0.0.0 to read on all network cards, 127.0.0.1 to limit to the current host).
- MQTT_OPTIONS:
  – port: TCP port of the MQTT server.
  – port: TCP port of the MQTT server.
  – host: IP address of the MQTT server.
  – clientId: ID client to show on MQTT server.
  – username: user name to connect to the MQTT server.
  – password: password to connect to the MQTT server.
  – clean: clean connection start.

## 4. Experimental Application Examples

This section shows the experimental results of Cross-PLC in various applications developed at the Ford plant in Valencia. In all of them, Cross-PLC integrates Rockwell and Siemens PLCs. In particular, it integrates Rockwell PLCs from the ControlLogix family (1756, 5555 series, Series 6x, Series 7x, Series 8x, and CompactLogix), and for Siemens, the S7 family compatible with ISO-on-TCP (some, for example, are S7-1200, S7-1500, S7-300, and S7-400).).

### 4.1. Mini-Term 4.0

**Figure 5:**
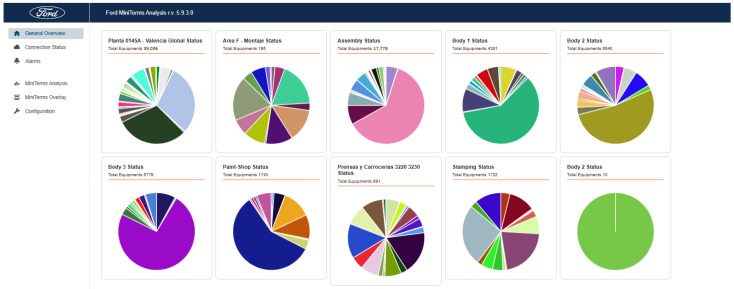
Distribution of Mini-terms installed in Ford factory (Almussafes, Valencia) 4.0. Connected elements.

**Figure 6:**
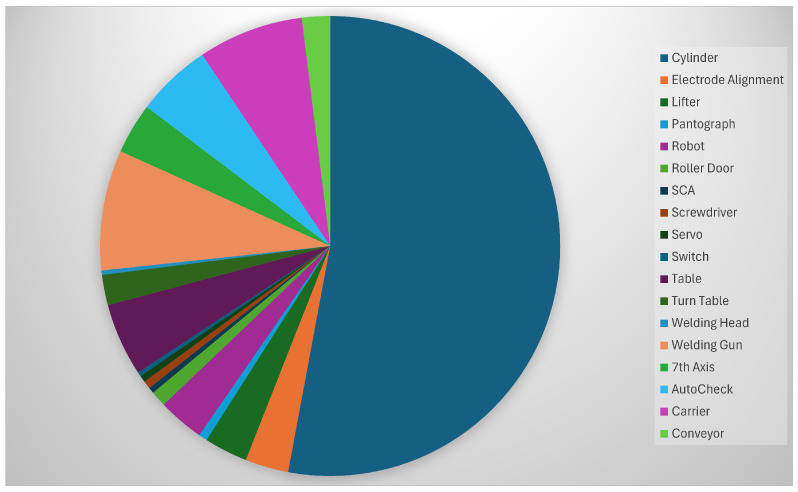
Elements monitored in body plant.

The Mini-term 4.0 application, see [22], is the most widely used today and is used to manage information using Cross-PLC. Figure 5 shows all the elements and components monitored by this platform. There are currently 39,036 monitored components. Figure 6 shows the details of the monitored components in the body plant. Elements like cylinders, lifter, pantograph, robots, roller door, screwdriver, motors, switch, welding guns, 7th Axis, etc., are elements that are under surveillance using Cross-PLC as a tool to extract the data using the IT/OT system installed in the factory.

### 4.2. Other I3oT Applications Developed Through Cross-PLCs

Since the Cross-PLC platform was put into operation, new I3oT applications have been developed to automate numerous tasks that before used to be carried out manually by technicians. The following sub-sections show some of them.

#### 4.2.1. Smart Call Solution

The first solution is to eliminate the large number of push-button switches (exclusive hardware with batteries) located in each of the plant lines that are used to send a reminder to the warehouse when material is required for the assembly of the product. The push buttons have been replaced by a call from the PLC that notifies the warehouse through the Cross-PLC when more material is needed, whether it is screws, gears, hinges, etc., or any mechanical elements that robots use to assemble parts. A new routine has been designed in the PLC that allows counting the number of times the work is carried out compared to the amount of material available on the line so that when the number of boxes of material automatically end, a signal is sent from the PLC to the warehouse to replace the boxes. With this, we will also be able to know the amount of material orders made daily and monthly and keep an exhaustive count of the parts produced.

#### 4.2.2. Automatic Production Line Status Monitoring

To carry out maintenance, repairs or any necessary action on the manufacturing lines, the first step is to remove the parts inside the line just before the stop. These gaps found in the line must be counted and calculated manually to subsequently communicate them to the previous processes so that the other lines are not blocked or full of parts, and thus, we will be able to perform the tasks safely and effectively, and therefore, at start-up, the lines will be filled again with the necessary material. Thanks to the Cross-PLC, this counting task has been automated, which allows more time to carry out the maintenance and start-up tasks of the line, improving these processes and above all, optimizing line stop preparation times.

### 4.3. Energy Consumption Monitoring

Another very useful tool developed has been the detection of work cells that are stopped or problems such as leaks in the supply systems by controlling the energy. In this case, we need to monitor the consumption of the different cells of a manufacturing area, and when an anomaly happens, it will be quickly detected where it is happening, reducing the time spent to solve the problems that may appear and improving energy efficiency.

## 5. Discussion

The presented tool, Cross-PLC, generates a significant improvement in information management when developing Industry 4.0 tools. As indicated in the introduction, there are numerous drawbacks to the massive implementation of IIoT tools in industry. The improvements generated by Cross-PLC from this perspective are described as follows:

- *Energy efficiency*: Since using the IT/OT network already available does not generate additional energy costs.
- *Interoperability*: Since it allows the interconnection and centralization of information available in different PLCs, making it transparent to the user.
- *Safety*: Since the Cross-PLC tool is only configured for data extraction, not for modifying production line information.
- *Scalability*: Since it does not present any limit on the brand of PLC or the control device to be connected.
- *Maintenance and updates*: Since using the devices already connected to the IT/OT network does not generate any additional maintenance or update costs.
- *IT/OT integration*: since the nature of the Cross-PLC proposal allows for this integration.
- *Cultural change*: Since for the user, and even for the developer, the IT part is opaque and does not generate any additional effort.

Communication between PLCs and crossPLC can be established in two main ways: natively, using the PLC’s own language, or through HTTP requests using JSON formats. Native communication, by using the PLC’s specific language, simplifies integration with that specific model. There are no issues with data translation or interpretation. However, compatibility between different versions of PLC firmware can be a challenge. If a new version introduces different data structures or commands, Cross-PLC would need to be updated to understand and interpret these changes. This requires constant maintenance of Cross-PLC to ensure compatibility with each version of each supported PLC model. The advantage is that performance is usually optimal as no format conversions are required. On the other hand, JSON-based communication offers greater flexibility and version compatibility. JSON is a lightweight and human-readable data exchange format widely used on the web. Its simple, standardized structure facilitates communication between different systems, regardless of the programming language or platform. This is the key to its robustness against version changes: as a PLC-independent data format, PLC firmware updates that do not modify the agreed-upon JSON structure do not affect communication with Cross-PLC. If new features or variables are added to the PLC, they simply need to be included in the JSON exchange without needing to modify the underlying structure. When interpreting the JSON, Cross-PLC would simply read the new data or ignore any data it does not need, maintaining compatibility. In other words, communication using JSON decouples the internal structure of the PLC from the Cross-PLC platform. While it is true that Cross-PLC does not support all PLC protocols, it does establish simple native communication for the main programmable controller protocols. For example, Rockwell only supports “unconnected” datagrams, but it simplifies PLC logic by allowing data to be transmitted as if it were another PLC. Currently, Cross-PLC supports http communication but it is only tested in Rockwell and Siemens PLC. Omron PLC also supports http communication but we have not tested it.

The *Cross-PLC* is compatible with other proposals, like, for instance, the new PLCs developed for Industry 4.0. such as IoT-PLC, see [19] or PLC 4.0, see [20] or the PLC for IIoT developed by the company Phoenix Contact, Germany. The *Cross-PLC* and then the *I3oT* concept only propose to use the installation setup first and then, when the installation setup is not enough, propose new devices

## 6. Conclusions

The creation of the Industrializable Industrial Internet of Things (I3oT) concept for the development of massively industrializable IIoT applications imposes the use of existing facilities in the company as a criterion. As seen in our previous work, this concept allows us to develop numerous tools capable of predicting a machine failure, detecting sub-bottlenecks and bottlenecks, rebalancing lines in real time, etc. The great drawback is that in order to continue maintaining the concept of I3oT, we need to use the IT/OT network to extract data from the PLCs installed in the factory. The Cross-PLC allows reading that information in a passive way with the PLCs sending the data when they have undergone a significant change. Cross-PLCs update the information in their memory, and the applications make use of this information for their operation using the Cross-PLC as a virtual PLC that provides it with information. This allows us, on the one hand, to extract plant information from the different PLC brands and, on the other hand, to standardize the I3oT application development process. In future works, we will continue with the research for new I3oT applications because the Cross-PLCs are very useful for solving the problems of communication through the IT/OT network of the factories.

## Data Availability

Data are contained within the article.

## Abbreviations

The following abbreviations are used in this manuscript:

IT/OT	Information technology/operational technology
